# Trust or robustness? An ecological approach to the study of auction and bilateral markets

**DOI:** 10.1371/journal.pone.0196206

**Published:** 2018-05-07

**Authors:** Laura Hernández, Annick Vignes, Stéphanie Saba

**Affiliations:** 1 Laboratoire de Physique Théorique et Modélisation, UMR8089 CNRS, Université de Cergy-Pontoise, Cergy-Pontoise, France; 2 Ecole Nationale des Ponts et Chaussées (ENPC), C.A.M.S.-EHESS, UMR CNRS 8557, Paris, France; 3 CRED- TEPP, Université Panthéon-Assas Paris II, Paris, France; Universidad Nacional de Mar del Plata, ARGENTINA

## Abstract

Centralized markets are often considered more efficient than bilateral exchanges because information is public and the same for all the agents. On decentralized markets, where the information is private, the influence of trust on the market outcome has been underlined by many authors. We present an empirical study of the distinctive Boulogne-sur-Mer Fish Market (where both buyers and sellers can choose to trade by either bidding or bargaining), focused on the interactions between agents. Our approach is inspired by studies of *mutualistic ecosystems*, where the agents are of two different types (as in plant-pollinator networks) and the interactions only take place between agents of different kinds, naturally providing benefits to both. In our context, where the two kinds of agents are buyers and sellers, our study shows that not only do their interactions bring economic benefits for the agents directly involved, but they also contribute to the stability of the market. Our results help to explain the surprising coexistence of the two forms of market in the distinctive Boulogne sur Mer Fish Market.

## Introduction

How do social interactions influence the outcome of economic exchanges? This question, which has generated long-lasting debate between economists and sociologists [1], is beginning to be addressed quantitatively by works focusing on the building up of trust in buyer-seller systems.

While it is clear that human beings rely on cooperation with others for their survival [2], economic literature mainly supports the idea that auction markets, which involve competition among buyers without any direct social interaction with the sellers, are the most informationally efficient way of organizing exchanges. The information is the same for all the actors and there is no possibility of arbitrage [3]. Following on, important efforts have been devoted to the development of auction theory, with a marked interest in the efficiency of the market [4]. More recent articles [5, 6] underline that, when goods are heterogeneous and there is no signal of quality, a decentralized mechanism (bilateral transactions) allows people to gather information and to better evaluate the intrinsic quality of goods. In this case, networks of social interactions play an important role in the exchange decisions [7, 8]. Then if, as has been stated in [9], networks underlie markets, it is essential to consider the robustness of the former as a necessary condition for the stability of the latter. In network theory, it is well known that the robustness of a given network in the face of errors or attacks is strongly related to its topology. We therefore concentrate our study on the statistical properties of the interaction networks of this market.

Most economic studies of markets traditionally deal with macroscopic variables such as the quantity and quality of exchanged goods, prices, and the comparative behaviour of different groups of agents. After some early works pointing at the relevance of networks in market organization [10–12], more recent studies have started to make full use of network theory to take into account the role of microscopic interactions: *who exchanges with whom?* [7, 8, 13–18].

The stability of markets is essential to the welfare of the population. In the case of food markets, this condition is one of the four pillars of food security [19]. Among other food markets, European fish markets are nowadays in a critical situation due to the scarcity of the resource, which has led to catch limits and quotas. The Boulogne-sur-Mer Fish Market, the most important in France in terms of quantity, is an excellent case study. This long-established daily market, which had operated historically in a decentralized way, was led by EU regulations to adopt a centralized structure. This new way of functioning was strongly rejected by both buyers and sellers, and in 2006 a double trading system was finally allowed to coexist in the same place. Every morning, when the buyers and the sellers (the fishermen) arrive on the market, they can choose to trade through an auction process or to exchange through pairwise transactions. The operating rules, the amounts and qualities of the goods exchanged, and the price distribution of both sub-markets have already been studied [8, 20, 21].

In this paper we present a comparative data-based study of the two sub-markets that focuses on the interactions between buyers and sellers. For several reasons, our dataset is ideal for our purposes. Detailed data concerning the daily transactions are available, allowing us to compare the behavior of agents in both sub-markets under similar economic, seasonal, climatic and social conditions, thus revealing the influence of social interactions on the economic strategies. The sample covers the period of reorganization, when the market split into a bilateral negotiation market and an auction market.

The observed persistence of both sub-markets raises the question of the conditions of their stability. In other words, if the auction market is more efficient, why did it not eclipse the bilateral negotiation market, leading to its extinction? On the other hand, why does the auction market continue to exist when most of the agents asked for its removal? One explanation could be related to the heterogeneity of the goods and a possible specialization of each sub-market (one selling high quality/high price goods and the other specialized in low quality/low price ones). However, this explanation does not seem to hold. In a previous article [20], it has been shown that most of the species (80 different species are traded at Boulogne-sur-Mer) are sold on both sub-markets and that the average prices are similar, even though the price distributions show that the the bilateral market is riskier than the auction one (higher variance and kurtosis). While some people (buyers or sellers) prefer one market to the other, others switch regularly between the two, so that on average, the relative quantities sold on each market remain fairly stable (40% on the auction market, 60% on the bilateral negotiation market, over the period studied).

In what follows, we investigate the influence of agents’ behavior and their social links on the stability of this organization. In the auction mechanism, the only action of sellers consists in choosing the minimum price at which they accept to sell. After this, they do not have any active role; the actual sale is handled by the auctioneer. He trades with the buyers, who compete against each other to buy the different goods. Decentralized exchanges, which allow pairs of non-anonymous buyers and sellers to discuss and bargain before trading through frequent matching (as it is the case in a daily market), can easily bring out trust and loyalty behaviors. We explore the influence of trust and the emergence of loyal behavior between buyers and sellers through empirical analysis, searching for stylized facts that might appear as distinctive features of each sub-market.

Interactions among agents can be mapped on a *bipartite* complex network, where the nodes are of two different kinds (in this market, people are either buyers or sellers) and the links, representing the interactions, can only connect nodes of different kinds.

Our data also allow us to build a bipartite network with *weighted links*, describing the number of contacts of any pair of agents during the period studied, for each market. One originality of this study is to adopt the tools developed to quantify ordering patterns in *mutualistic* systems in ecology, in order to describe the differences in the market allocations coming from different price mechanisms. Of course, this does not mean that these markets necessarily follow the same constitutive dynamic rules as ecosystems. A similar approach has been applied to the study of import-export relationships and industrial ecosystem networks [15–18].

In mutualistic ecosystems like plant-pollinator or plant-seed disperser networks, the interactions between two agents of different types naturally brings benefits to both of them. The pattern of interactions observed in such systems is far from random. Instead, it displays a particular structure called *nestedness*. This particular topology of the network is such that if the columns (rows) of the bipartite adjacency matrix are ordered in decreasing or increasing degree, then the rows (columns) appear to be ordered in the same way. Thus, when species are ordered, for example, in decreasing degree, the contacts of a given species constitute a subset of the contacts of the preceding ones, thus leading to an adjacency matrix where all the contacts are located in a corner [22, 23] (see S1 Appendix for details). We investigate whether a similar pattern, revealing some degree of organization (as opposed to a uniform distribution of contacts), is observed in either of the sub-markets studied.

A parsimonious analysis of network properties shows that the structures of the social interactions involved in each sub-market are different. In order to detect a signal of trust in our data, we define a *loyalty index* measuring the frequency of the interactions between the different pairs of agents in each market organization. The distribution of this loyalty index clearly depends on the type of sub-market: while it appears to be scale-free in the decentralized (bilateral negotiation) sub-market, it shows a sharper decrease in the auction market, suggesting that for the latter, there is a specific value of loyalty above which the number of pairs with higher loyalty falls very fast.

## Materials and methods

### Description of the data-set

A detailed electronic register of daily transactions on the Boulogne-sur-Mer Fish Market has been kept since 2006, when the coexistence of the two sub-markets started. For each transaction, the identification of the buyer and seller involved, the amount and quality of the goods exchanged and the transaction price are recorded. This work is based on data collected during eighteen months in 2006 and 2007, thus covering the reorganization period.

A seller *i* and a buyer *j* may make several transactions on the same day (they may exchange different lots of fish at different prices). In this study, following [20], we coarse-grain daily transactions, and we consider that a seller *i* and a buyer *j* hold one *contact* on day *d* if they perform *at least* one transaction on that day, regardless of the price or the quantities exchanged. Accordingly, the number of contacts of a pair of agents in a given period corresponds to the number of days when they had at least one contact.

### The model

From the previously described database, we build a network of interactions which can be coded into a *simple* bipartite matrix, *K*, whose elements *K*_*i*, *j*_ ∈ [0, 1] indicate whether or not the seller *i* and the buyer *j* have interacted during the selected period, and the *weighted* bipartite matrix, *B*, of elements $B_{i,j}\in N$ which reports the number of contacts during the period.

For this work, we have chosen not to include a weighting for the links, which would take into account information about the prices or the quantities exchanged. This is certainly a simplifying hypothesis, but it also serves another purpose. As this market is very heterogeneous (with big and small buyers and sellers) this assumption is needed to prevent the information about social interactions from being blurred by the different buying/selling capacities of the agents involved. Previous results [20] confirm that this hypothesis still allows for the comparison we are interested in, by showing that on average, prices are not significantly different on the two sub-markets.

The degree of an agent, ($k_{i}^{S}=\sum_{j}\;K_{ij}$ for sellers and $k_{j}^{B}=\sum_{i}\;K_{ij}$ for buyers) gives the number of *different* customers (suppliers) that a seller (buyer) has in the chosen period. The strength of an agent, ($s_{i}^{S}=\sum_{j}\;B_{i,j}$ for sellers and $s_{j}^{B}=\sum_{i}\;B_{i,j}$ for buyers) gives the total number of contacts that the agent has during the period, regardless of the counterparts with whom the agent has these contacts. By comparing the two, we can find out whether the strength of a seller (buyer) comes mainly from contacts with different buyers (sellers) or from repeated contacts with a smaller, preferred group of buyers (sellers).

Nestedness is an indicator of some degree of structure in the network’s links, which is commonly observed in mutualistic ecosystems. It is different from a random uniform distribution of contacts and reflects a very particular form of organization. It highlights two different behaviors among the agents of each type (buyers and sellers). Some of them are generalists, having (many) contacts with their counterparts, while others are specialists, having contacts mainly with generalist counterparts and rarely with specialists. Therefore, nestedness is not only different from a random structure of interactions, but also different from another common ordered state described by a block type matrix, which would indicate a niche structure of the market.

The bipartite matrix of a perfectly nested network, when conveniently ordered in decreasing degree of one type (buyers or sellers), shows a triangular shape, with all its zero and non-zero elements on either side of a curve called *extinction curve* or *isocline of perfect nestedness* (IPN) that can be analytically determined as a function of the number of rows, *n*, the number of columns, *m*, and the density of contacts, *ϕ* [24] of the bipartite matrix *K* (see S1 Appendix for further details).

Several indicators aimed at quantifying nestedness coexist in the ecological literature. They are based on different properties of a nested system, and have different advantages and drawbacks. In order to avoid bias, here we measure the nestedness of both sub-markets using four well known indices, each one targeting a different property of the nested matrix. We use the indices proposed in the NED [25] package, along with an alternative measure of nestedness based on the resistance of the network to targeted attacks. If some species of one type are eliminated from the system, then the counterparts that no longer have any contacts will also disappear (secondary extinctions) and eventually the whole system may collapse. In what follows, we measure the robustness of a system as its capacity to resist such attacks. The NIR, *nesting index based on robustness* of the network [26] exploits the fact that a nested system reacts very differently to attacks consisting in the suppression of nodes of one type in increasing or decreasing degree order (see S2 Appendix for more details).

An indication of trust may be obtained by measuring the frequency of contacts between any two agents. We define a *loyalty matrix* based on the weighted bipartite matrix *B*:

$$\begin{array}{c} L_{i,j}=\frac{2\times B_{ij}}{s_{i}+s_{j}} \end{array}$$ (1)

Note that *L*_*i*, *j*_ = 1 if seller *i* and buyer *j* only transact between themselves and *L*_*i*, *j*_ ≪ 1 if seller *i* and buyer *j* transact much less between themselves than with others agents in the same period.

## Results

We analysed the properties of the *simple* bipartite matrix, *K*, and the *weighted* bipartite matrix, *B*, both in a global and in a detailed time scale, by studying the networks obtained for both sub-markets, using not only the data integrated over the whole period (18 months) but also differentiating between seasons and between days. This multi-scale study is necessary because data integrated over the whole period lead to matrices of larger size, allowing for the study of the statistical properties of the system, while zooming in on chosen periods (from seasonal to daily) allows to retrieve information about the structure of interactions that could have been washed out by the coarse-graining procedure.

Focusing on the structure of the interactions between agents brings to light valuable information about the agents’ behavior in each sub-market.

### Degree distributions: The number of partners

The degree of a seller (buyer) indicates the number of different customers (suppliers) this agent has.

**Fig 1:**
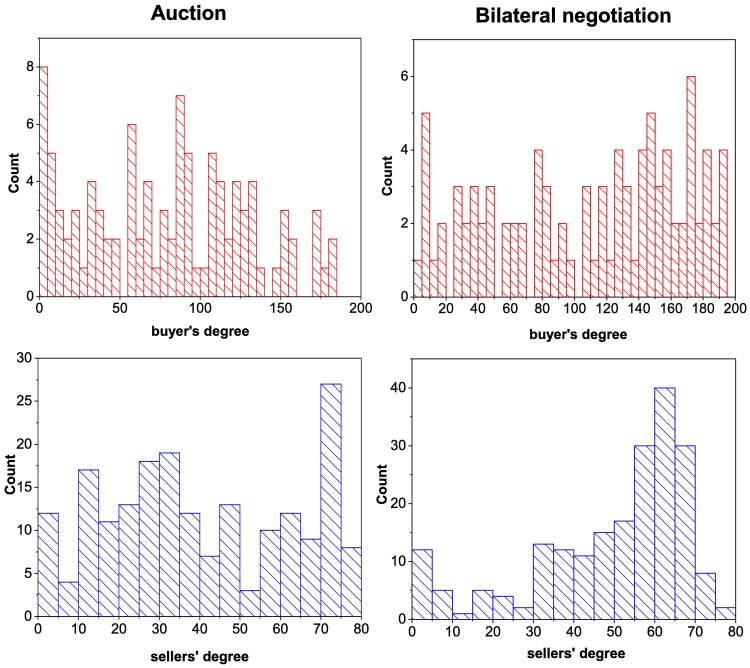
Degree distributions for both sub-markets. Left auction, right bilateral negotiation. Top panels, buyers’ degree, bottom panels sellers’ degree.

Fig 1 compares the degree distributions of buyers and sellers for the two sub-markets. The degree distribution for buyers (top) does not show any particular structure in either of the two sub-markets, looking like a uniform distribution with large fluctuations. However, high degrees are more frequent in the bilateral negotiation sub-market, which may be understood as a signature for the *exploration phase* of the buyers. In fact, when buyers enter this sub-market they diversify their suppliers before choosing their preferred partners. This exploration phase will appear later through the comparison between degree and strength distributions.

Conversely, the sellers’ degree distributions (bottom) of the two sub-markets are very different. The bilateral negotiation sub-market shows a left-skewed distribution, with a maximum of around 60 customers, showing that about half of the sellers have many different customers, while for the auction market the distribution seems quite uniform with large fluctuations. This reflects the fact that in the auction market, sellers cannot influence the transaction prices once the minimum price for their goods has been fixed, while in the bilateral sub-market, they can adapt their strategy to attract more customers.

### Strength distribution: The number of contacts

The strength of a seller (buyer) measures the number of contacts that this agent has had during the period studied (with the same or different counterparts). In order to understand the following graphs, a rough estimation of the maximum number of transactions is helpful. In these markets there are typically 200 sellers and 100 buyers, and the full period covers 18 months, which is about 450 working days (the market does not operate on Sundays). So a seller who was present on every market day and dealt with all the buyers every day would have a degree of $k_{s}^{MAX}\approx 100$ and strength of $s_{s}^{MAX}\approx 100\times 450=45000$. The strength values observed are much lower because the agents are not present in the market every day.

**Fig 2:**
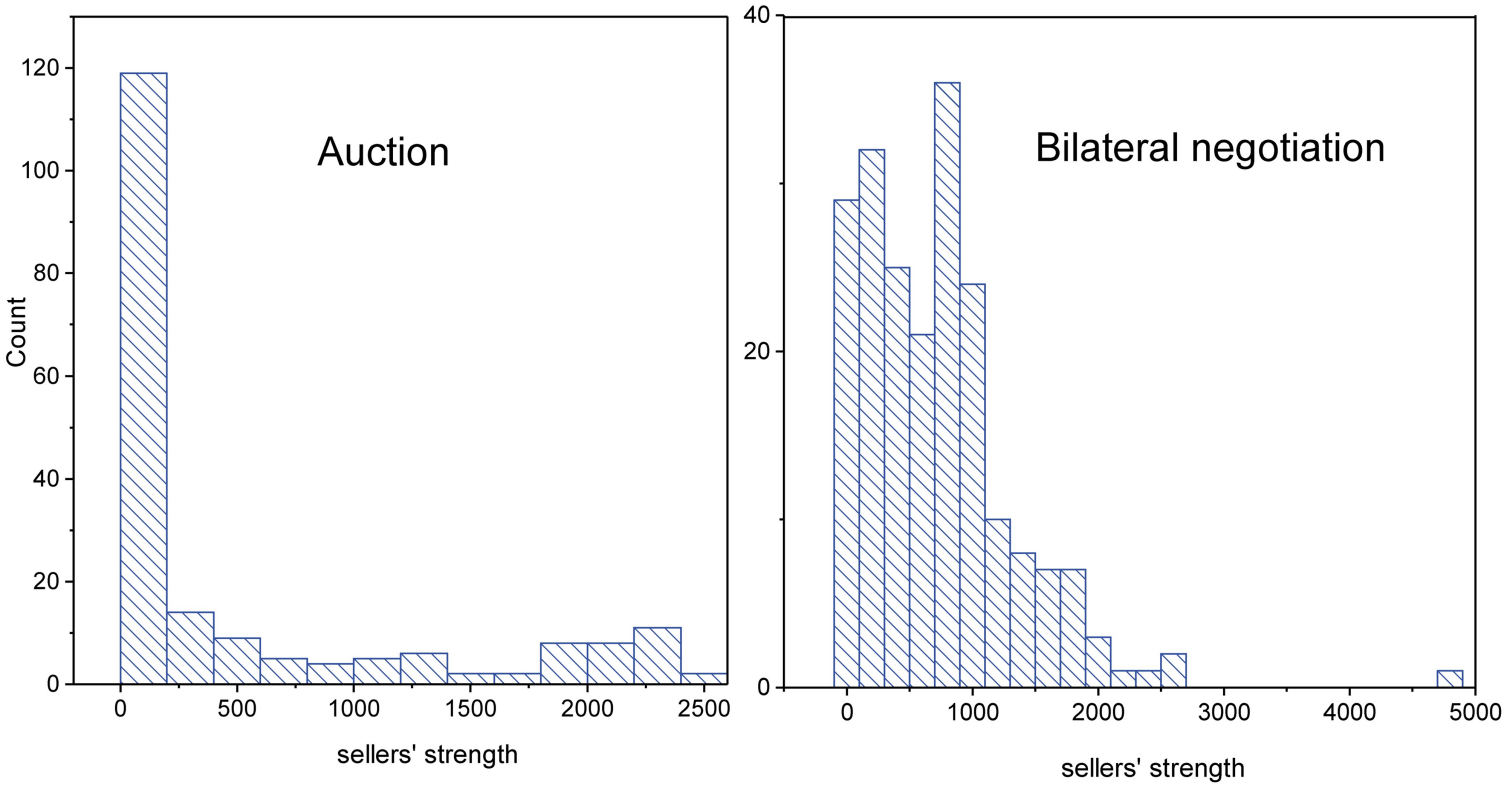
Sellers’ strength distributions for both sub-markets. Left: auction; right: bilateral negotiation. Bin size is the same (200) for all the panels.

The comparison of sellers’ strength distributions in the two sub-markets shown in Fig 2 is particularly interesting. While it is uniform in the auction sub-market (except for a peak at a very low number of transactions), the distribution corresponding to the bilateral negotiation sub-market is Poisson-like (if we exclude very low strength values), with a large fraction of sellers who have many transactions during the period. Comparison with the values given by the degree distribution of sellers reveals that instead of diversifying their customers, on average, they trade repeatedly with the same ones.

Buyers’ strength distributions, which are monotonously decreasing with degree, do not show any differences between the sub-markets, except for the fact that the tail is much longer in the bilateral negotiation sub-market (see Fig A in S3 Appendix).

**Fig 3:**
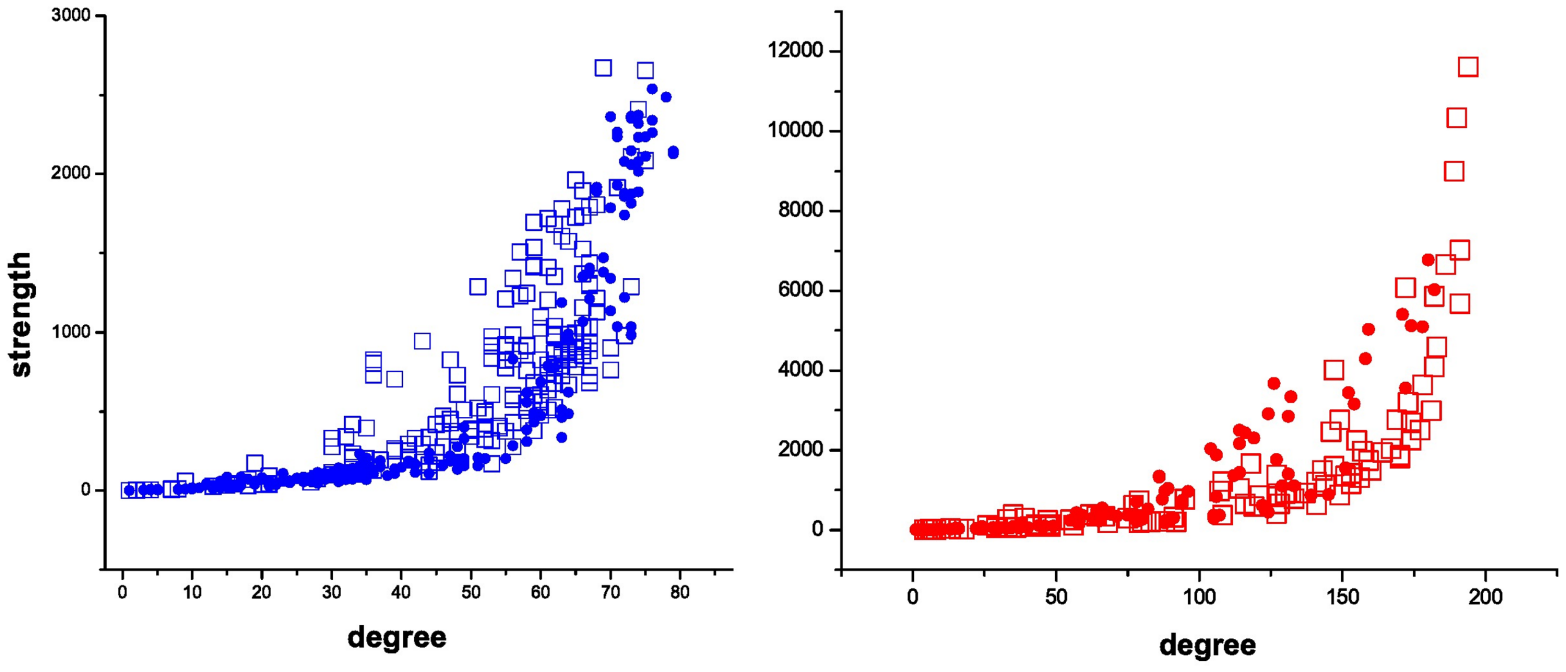
Strength-degree correlation. Open squares: bilateral sub-market market; full circles: auction sub-market. (a)Left panel: sellers; (b)right panel: buyers.

Fig 3 shows the scatter plots of the strength vs. the degree correlation in each sub-market. The sellers’ curve grows faster in the bilateral negotiation sub-market than in the auction sub-market, indicating that the sellers have loyal customers that come repeatedly to transact with them. The buyers’ curve is also interesting because it reveals there is an “exploratory” phase in the bilateral sub-market, where the buyers multiply contacts with different providers (high degree and not so high strength) while looking for those that are the most suitable for their needs.

### Loyalty index: A signature of trust

To better understand the different results obtained for the two sub-markets, it is interesting to consider the intrinsic differences in the social relations between the agents in each sub-market. The loyalty matrix defined in Eq 1 captures these differences. In the auction sub-market, the buyer-seller interaction is mediated by the auctioneer, while in the decentralized sub-market, buyers can discuss directly with the sellers about the quality of the goods and the conditions of the exchange. Here, buyers seek the trading conditions that best suit them, through the experience they obtain from repeated exchanges.

**Fig 4:**
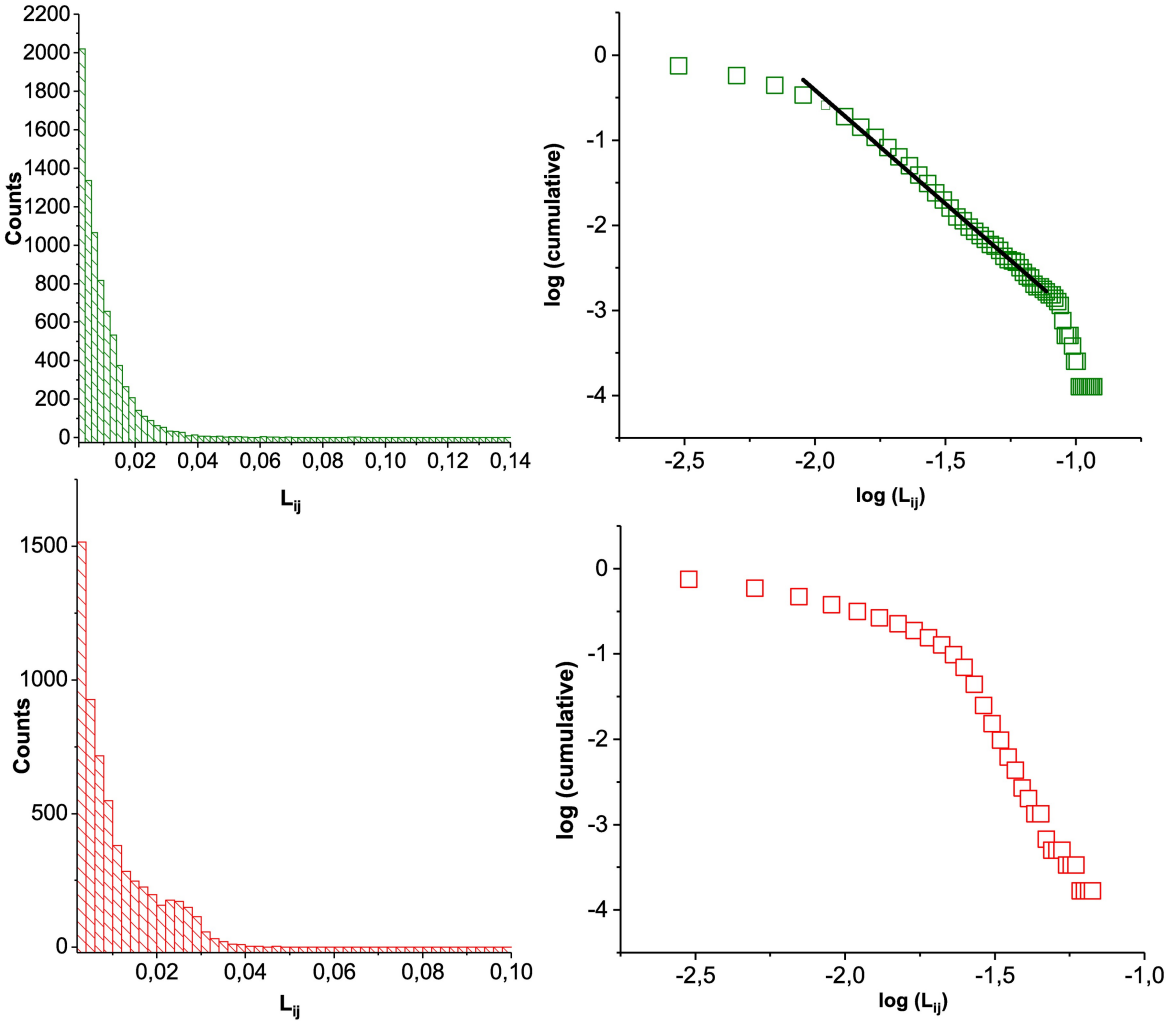
Loyalty index. Negotiated (auction) sub-market data is shown in upper (lower) panels. Left panels: loyalty distribution; right panels: cumulative loyalty distribution.

Fig 4 (left panels) shows the distribution of the loyalty for both markets. The bilateral negotiation sub-market has both a larger tail and a larger number of pairs with a small loyalty index (50% over the auction market). These curves are measured over the whole period, so the excess of pairs with a small loyalty index in the negotiated market is again a signature of the “trials” performed by buyers when they first join the market and the longer tail comes from the pairs that establish as trading partners in the long run. These repeated interactions may also help the sellers to adapt their strategies.

Interestingly, the cumulative distributions of loyalty in these sub-markets (right panels) clearly reveal qualitatively different behavior. In the auction market, there are two different regimes: the cumulative number of pairs having low and average loyalty values varies slowly until a critical value where the probability of finding pairs with a higher loyalty decreases very fast. In the bilateral market, on the contrary, there is no such characteristic critical value. This lack of characteristic scale suggests that this cumulative distribution could be fitted by a power law (with a finite-size cut-off). However, the data range is too short for the actual value of the exponent (which we found to be *α* = 2.66 ± 0.03) to be meaningful. In spite of this limitation, which is true for both sub-markets, the important result here is the clear qualitative difference between the shapes of these two cumulative distributions.

### The nested structure of bipartite matrices

**Fig 5:**
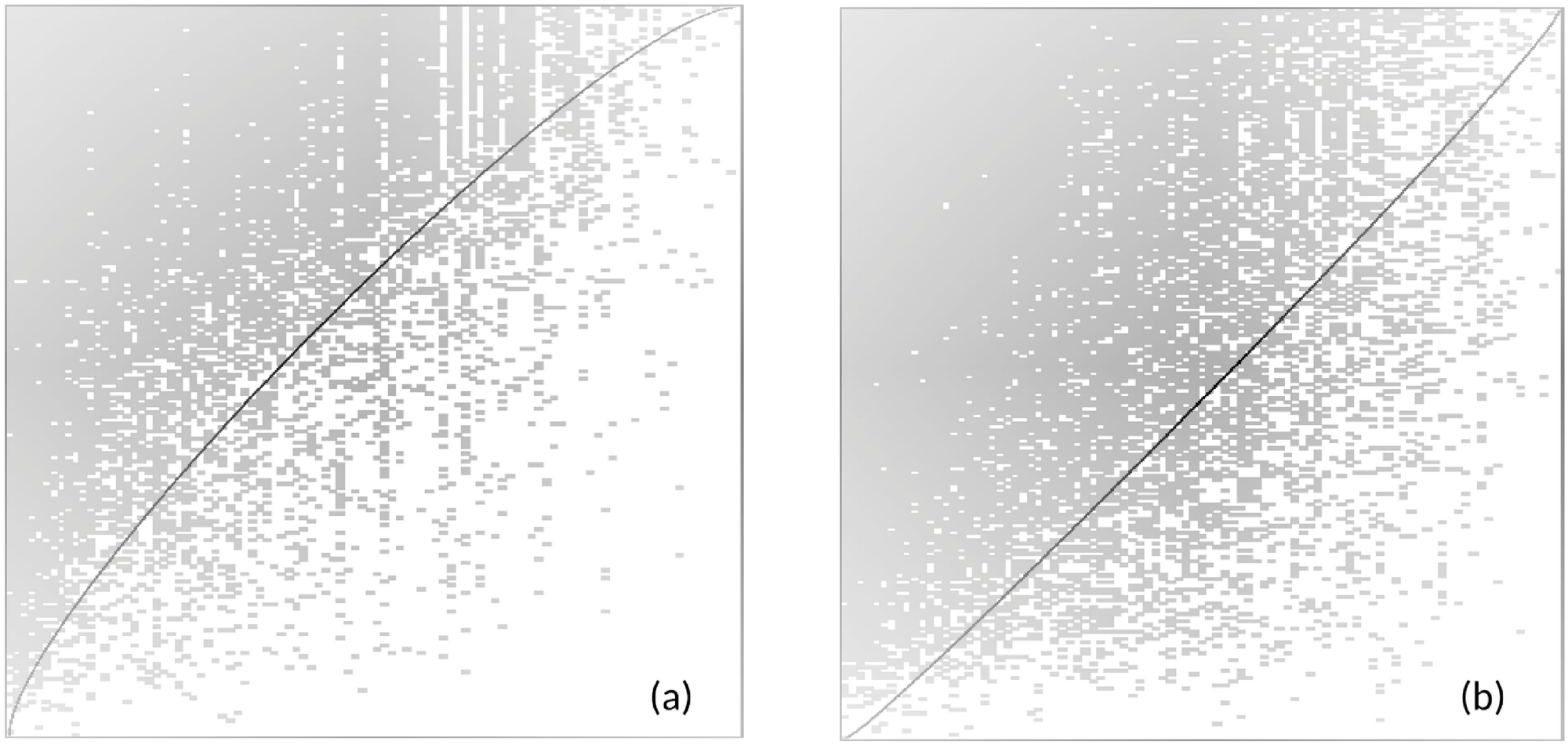
Bipartite interaction matrices for both sub-markets. Sellers in rows, buyers in columns, ordered with the highest degrees of rows and columns on top and left respectively. Black dots correspond to *K*_*ij*_ = 1. (a) Left panel: auction market; (b)right panel: negotiated market.

In mutualistic ecosystems, the observed nested structure of the bipartite matrices describing the network is known to contribute to their robustness and stability [27]. We have analysed the *K* matrices of both markets using different metrics developed for the study of ecosystems, noting however, that the densities of contacts of both markets measured over the whole period (*ϕ* ≈ 40 – 50%) are much higher than those typical of mutualistic ecosystems, which are much sparser. Fig 5 shows the bipartite matrices *K* of both sub-markets for the whole period studied. They are ordered so as to bring to light the nested structure (if any). Both types of agent (buyers and sellers) appear ordered by decreasing degree, with the maximum degree on the top-left corner. The resulting structure clearly shows that the interactions are not randomly distributed in a uniform way in either of these sub-markets.

All the nesting indices show an important degree of nestedness in both sub-markets, thus revealing the existence of generalist buyers and sellers along with some specialists of both types who mainly interact with generalists (see Tab.A in S3 Appendix). This fact also excludes a *block type* adjacency matrix, which would correspond to a market segmented into “niches” of interaction among groups of buyers and sellers who only interact between themselves. Although the existence of some groups of specialized buyers and sellers cannot be excluded, they are not dominant in the structure of interactions of these markets.

We have also studied daily matrices using the nesting index based on robustness described in [26]. This index studies how the system reacts to two extreme targeted attacks. In the attack strategy called *decreasing degree removal* (DDR), agents of one type (either buyers or sellers) are removed in order, starting from those of highest degree, and the agents of the other type who are left without any contacts are considered to disappear from the market (secondary extinctions). The attack tolerance curve (ATC) gives the fraction of agents of one type that remain in the market as a function of the fraction of agents of the other type who are removed. The extreme opposite attack strategy, called *increasing degree removal* (IDR), consists in the same procedure, but now the agents to be removed are chosen in increasing degree order. This attack strategy is less sensitive to the network ordering [26] (see S2 Appendix for details).

**Fig 6:**
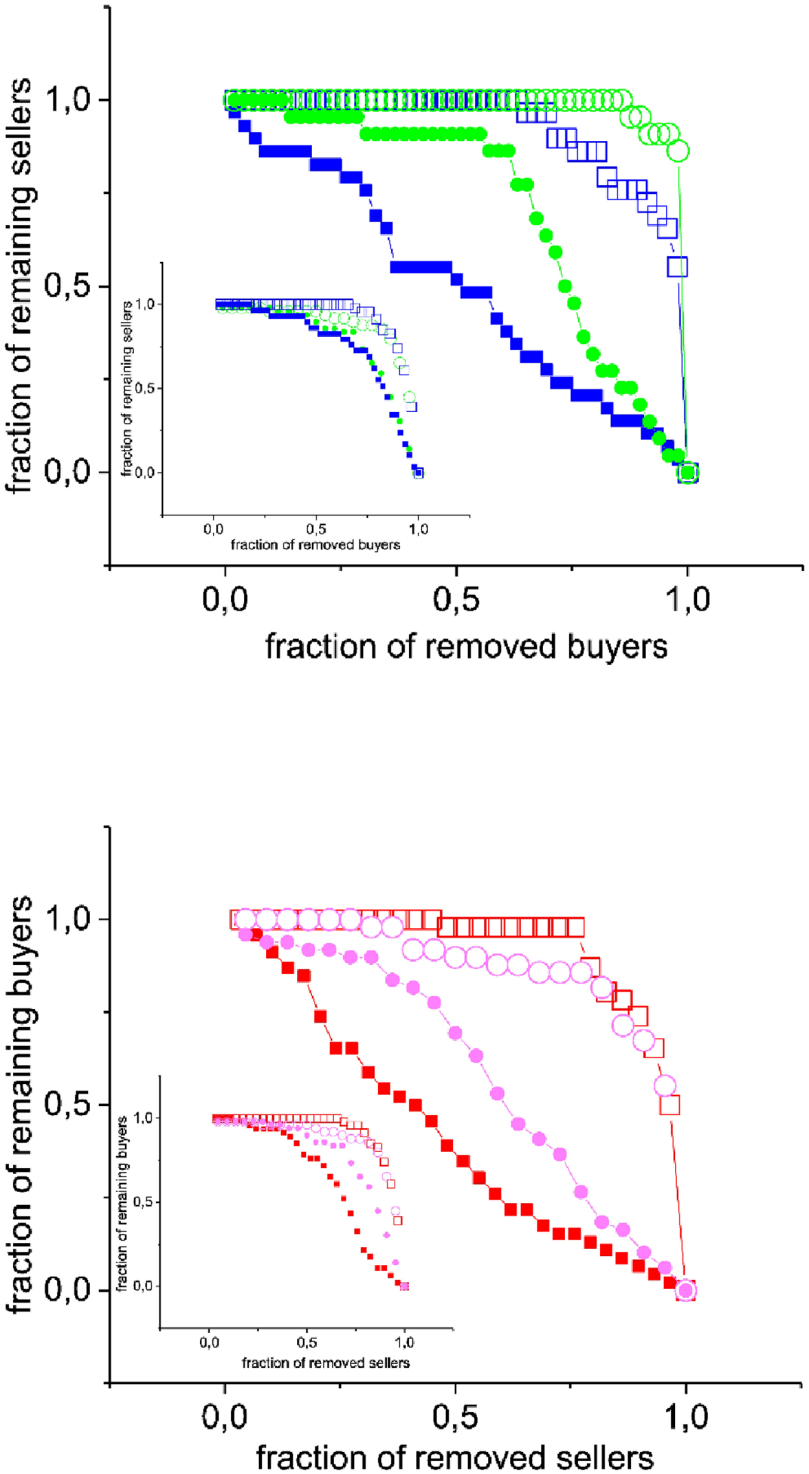
Attack tolerance curves (ATC) for auction and negotiated markets. Data corresponding to Friday, week 16, April, 2007 (Fridays are days of high activity in the market). (a)Upper panel: ATC for sellers. Open symbols correspond to the IDR strategy, and full symbols to the DDR strategy. Squares correspond to negotiated market data and circles to auction market data. (b)Lower panel: ATC for buyers. Same symbols as in upper panel. In the insets we depict, with the same notation, the ATC curves for the randomized version of each network (same size and fill), after 1000 randomization steps (ATCs for data corresponding to other Fridays of April 2006 and 2007 can be found in S3 Appendix).

Fig 6 (upper panel) compares the fraction of sellers who remain in the market when buyers are removed applying either the DDR or the IDR strategy.

We can clearly observe that for the DDR strategy (full symbols), the ATC of the bilateral market decreases faster than the ATC for the auction market as buyers are removed. The area between the ATCs corresponding to the two attack strategies, which is proportional to the nesting index, is larger in the case of the bilateral sub-market (see Fig B and Fig C in S3 Appendix for details).

The lower panel of Fig 6 shows the ATC for buyers. Again we observe that the area between the curves corresponding to the two attack strategies is larger for the bilateral market, thus giving a larger nested index for buyers in this market as well.

Interestingly, for the auction markets the ATC for sellers decreases very slowly even for the DDR strategy, indicating that an important fraction of the buyers have to be removed before a significant effect is observed on the sellers. Moreover, the DDR ATC curves for sellers are step-wise, while those for buyers are not (lower panel). This means that when high degree buyers are removed from the market, most of the sellers may still find customers, while as sellers disappear, buyers are immediately affected. This effect is dominant in the auction sub-market, where a removal of about 50% of the generalist buyers affects the sellers very little. This observation suggests that the auction market is more robust to fluctuations than the negotiated market, which could be an argument in favour of auction.

As the density of contacts in these sub-markets is much higher than in mutualistic ecosystems, the sensitivity of this nesting indicator is diminished. However it should be noted that in all cases, the nesting coefficient (cf. the area between the IDR and DDR curves) is much larger than the one corresponding to randomized matrices (in the insets) of the same characteristics, thus indicating again that the structure of interactions is not random.

## Discussion

We have performed a data-based study of the interaction networks of the two coexisting sub-markets (auction and bilateral negotiation) of the Boulogne-sur-Mer Fish Market, searching for stylized facts characterizing agents’ behavior and market structure. By applying tools drawn from the study of mutualistic ecosystems, we observe that both sub-markets are nested, although typical market densities are much higher than those observed in mutualistic ecosystems. It is noteworthy that the densities of contacts corresponding to the whole period and to daily data are quite different. The density is quite high for both markets when the whole period database is analyzed (50% for the bilateral market and 40% for auctions), but it is much lower, and with a reverse tendency, for daily matrices (10% and 20% respectively), thus affecting the nesting indices. In other words, at a daily level, the proportion of pairs trading on the auction market is larger than in the bilateral market: however, when data are analyzed for the whole period, the bilateral sub-market has more trading pairs. While it is easy to understand the aggregation of dense matrices producing a denser matrix (the auction case), how can we explain that the aggregation of sparse matrices might lead to an even denser one? This paradoxical result can be understood by recalling that the degree distribution of buyers in the bilateral sub-market exhibits a longer tail than in the auction market, revealing that a “search phase” takes place every time a new buyer arrives in the market, thus increasing the density of the matrix aggregated over the whole period.

Moreover, we observe that the daily *K* matrices of the bilateral sub-market are larger than the corresponding matrices of the auction sub-market and comparable in size to the *K* matrices obtained for the whole period. This means that each day, the affluence in the bilateral sub-market is higher (the corresponding matrices are larger) but the number of trading pairs is smaller (the corresponding matrices are sparser). The observed fact that the *K* matrix corresponding to the whole period of the bilateral sub-market (obtained by overlapping daily matrices) is denser than the corresponding daily matrices necessarily implies that in this sub-market, the trading pairs are mainly different from day to day, thus increasing the number of ′1*s*′ in the aggregated matrix.

The ATC curves reveal that the auction market is the more robust. Almost 50% of the high degree buyers can be removed in daily matrices without significantly affecting the fraction of sellers that still have at least one contact. Certainly, this does not mean that removing those buyers is innocuous for the auction market, as it may affect volumes and prices. The centralized structure of the information helps the crossing of supply and demand. The adjustment will be performed by the prices. For the negotiated market, removing the same fraction for buyers leaves a larger fraction of sellers without any contact, thus revealing the importance of social relationships between agents. As sellers are fixed for the day, if their loyal buyers are absent, due to market frictions (search costs, asymmetry of information), it is costly for the sellers to match new customers.

The defined loyalty index measures the proportion of contacts between a given pair of agents with respect to the total amount of contacts of both, as an indicator of the trust existing in that pair. Its cumulative distribution shows two regimes for the auction sub-market: it decreases slowly for low values of loyalty, until a critical value beyond which it decreases very quickly. On the contrary, the distribution of loyalty in the bilateral sub-market looks like a power law with no typical scale of the loyalty index.

It is worth recalling that the results discussed here are based on networks whose links only take into account social behaviour, disregarding the prices and the quantities exchanged for each pair. As stated in the Methods section, this assumption, based on the results of previous work which show that on average, prices and quantities are not a discriminant of sub-markets, is necessary to study the role of social contacts in the formation of trust. Weighting the links with the information on prices and quantities exchanged may blur the phenomenon we want to measure. In this very heterogeneous market where agents have very different trading capacities, if for example, a big and a small buyer both buy from the same seller, the fact of weighting the links by the quantities exchanged or the price paid might lead to the weight of the corresponding interactions becoming comparable even if the big buyer interacts only rarely with the seller while the small buyer comes to trade repeatedly with him. The next step will be to build networks which include the information about price and quantity, which, along with the results presented here, may bring new information about different aspects of this system. This work is in process.

## Conclusion

In spite of the widely accepted view that auction markets are more efficient than bilateral ones, the case of the Boulogne-sur-Mer Fish Market, where there is no signal of quality for the goods, shows that both market organizations can coexist without one eclipsing the other. Our study provides a measurement of social interactions which sheds light on how they reinforce trust in the bilateral market, thus resulting in mutual benefit for the traders. This mutual benefit seems to compensate for the lack of information and the cost of linking. Our analysis also provides empirical evidence supporting the performance of the auction market, which is more resistant to the extreme case of the absence of high degree buyers. By focusing on social interactions, we can measure the global properties characterizing each sub-market which help to explain their observed coexistence. Our results suggest that the advantages offered by each sub-market help to increase the stability of the whole.

## Supporting information

S1 Appendix SI to introduction, nestedness definition. Description of a perfectly nested mutualistic ecosystems.
(ZIP)

S2 Appendix SI to materials and methods. Description of different nestedness indices.
(ZIP)

S3 Appendix SI to results. In this supplement we give the nestedness values obtained with different indices for the global period along with details of the daily analyzed matrices and their nestedness index using NIR. We also provide more examples as a complement of the results shown in Figs 2 and 6 of the main text.
(ZIP)
